# Polyunsaturated fatty acids ameliorate aging *via* redox-telomere-antioncogene axis

**DOI:** 10.18632/oncotarget.14236

**Published:** 2016-12-26

**Authors:** Jingnan Chen, Yan Wei, Xinyu Chen, Jingjing Jiao, Yu Zhang

**Affiliations:** ^1^ Department of Food Science and Nutrition, College of Biosystems Engineering and Food Science, Zhejiang University, Hangzhou, Zhejiang, China; ^2^ Department of Nutrition, School of Public Health, Zhejiang University School of Medicine, Hangzhou, Zhejiang, China

**Keywords:** polyunsaturated fatty acids, anti-aging, redox, telomere protection, antioncogene, Gerotarget

## Abstract

Polyunsaturated fatty acids (PUFA), a group of nourishing and health-promoting nutrients, ameliorate age-related chronic diseases. However, how PUFA especially n-3 PUFA exert anti-aging functions remains poorly understood. Here we link fish oil, docosahexaenoic acid (DHA) and arachidonic acid (AA) to the aging etiology via a redox-telomere-antioncogene axis based on D-galactose-induced aging mice. Both fish oil and PUFA enhanced hepatic superoxide dismutase (SOD) and catalase activities and cardiac SOD activities within the range of 18%-46%, 26%-65% and 19%-58%, respectively, whereas reduced cerebral monoamine oxidase activity, plasma F_2_-isoprostane level and cerebral lipid peroxidation level by 56%-90%, 20%-79% and 16%-54%, respectively. Thus, PUFA improve the *in vivo* redox and oxidative stress induced aging process, which however does not exhibit a dose-dependent manner. Notably, both PUFA and fish oil effectively inactivated testicular telomerase and inhibited *c-Myc*-mediated telomerase reverse transcriptase expression, whereas n-3 PUFA rather than n-6 PUFA protected liver and testes against telomere shortening within the range of 13%-25% and 25%-27%, respectively. Therefore, n-3 PUFA may be better at inhibiting the DNA damage induced aging process. Surprisingly, only DHA significantly suppressed cellular senescence pathway evidenced by testicular antioncogene *p16* and *p53* expression. This work provides evident support for the crosstalk between PUFA especially n-3 PUFA and the aging process via maintaining the *in vivo* redox homeostasis, rescuing age-related telomere attrition and down-regulating the antioncogene expression.

## INTRODUCTION

Aging is defined as the progressive decline of biological function with an increasing risk for many late-life onset diseases, including cancer, diabetes, neurodegeneration and metabolic syndromes [1]. As a major elucidation that underlies aging, excess reactive oxygen species (ROS) and induced oxidative stress adversely cause inevitable damage to organisms and age-related pathological conditions. This comprises the principle of the well-known free radical theory of aging [2]. Mice administered with oral or subcutaneous D-galactose, a reducing sugar that generates advanced glycation end products *in vivo* [3], are extensively used to mimic the aging process. This oxidative damage-induced aging model is accompanied by high-level thiobarbituric acid reactive substances (TBARS), low superoxide dismutase (SOD) activity in various tissues [4, 5], telomere loss and compromised telomerase activities in the hippocampus [6].

Accumulating evidence has clarified the change of telomeres in human aging-related diseases and the aging process [7] while telomere attrition is acknowledged as a robust hallmark of aging [8]. Telomeres, which are characterized by repeated DNA sequences at the terminal end of eukaryotic chromosomes, are protective against the DNA damage response and are essential for genome stability [9]. Unfortunately, telomeres naturally shorten during cell division, which ultimately triggers replicative senescence [10]. The telomere length is canonically maintained by a ribonucleoprotein reverse transcriptase referred to as telomerase [9]. In humans, telomerase is only ubiquitously expressed during the initial weeks of embryogenesis, followed by down-regulation in most cell types. Therefore, the inactivated telomerase activity and telomere attrition function as a tumor-suppressing mechanism by preventing cells from dividing indefinitely [10].

Both p53-p21 and p16-pRb are major cellular pathways during the senescence process. The *p16* expression, which markedly increases with aging in many tissues in rodents and humans, may be used as a biomarker of physiologic age [11]. Physiological *p53* activity is beneficial for cancer prevention and aging protection, whereas excessive *p53* activation is detrimental to healthy aging [12]. Increasing *p16* and *p53* levels are commonly induced in senescent cells and have been identified as consistent oncogene-induced senescence markers both in humans and mice [11, 13].

Nutrition is believed to promote healthy aging. In this context, n-3 polyunsaturated fatty acids (PUFA) are promising as an anti-aging dietary supplement. Representative n-3 PUFA bioactive compounds include docosahexaenoic acid (DHA) and eicosapentaenoic acid (EPA) which are abundant in fish oil. These primarily marine-derived fatty acids are able to ameliorate chronic diseases and many age-related diseases or impairments [14, 15]. Moreover, recent studies have shed light on the association between n-3 PUFA and senescence. For example, DHA prevents tumor necrosis factor-alpha (TNF-α)-induced senescence and dysfunction in endothelial cells [16], while concentrated fish oil extends the lifespan of lupus-prone short-lived (NZB×NZW)F1 mice [17]. However, the mechanisms responsible for n-3 PUFA counteracting senescence remain poorly understood. Moreover, several critical studies have demonstrated the association of n-3 PUFA with the delay of human telomere shortening. A 5-year follow-up study reported an inverse relationship between the baseline levels of whole blood n-3 PUFA and the rate of telomere shortening in 608 ambulatory outpatients with stable coronary artery disease [18]. Another work indicated that telomere shortening in elderly individuals with mild cognitive impairment or patients with chronic kidney disease may be attenuated with n-3 PUFA supplementation [19]. To our best knowledge, no animal experiment has been conducted to investigate the mechanisms of telomere protection by n-3 PUFA because the functionality of n-3 PUFA remains emerging in the anti-aging field, let alone its influence on telomere.

Here we systematically investigated the anti-aging effect of fish oil and long-chain PUFA monomers on D-galactose-induced mice in facets of *in vivo* redox-telomere-antioncogene axis. We subsequently emphasized the effect of PUFA on oxidative stress in aging mice and evidenced the PUFA protection of telomere and antioncogene homeostasis *via* comparing the functionality of n-3 and n-6 PUFA.

## RESULTS

### Body weights

There was no significant difference of body weights among all groups at the baseline levels. However, the weights of mice in the aging model group significantly decreased due to the aging outcome compared with those in the saline control group at the end of animal study (*P* < 0.05). Nevertheless, body weights were not significantly changed in all other groups of mice induced by D-galactose treatment (*P* > 0.05) except the moderate-dose No. 2 fish oil (200FO2) group (Supplementary Table S1).

### PUFA improve the *in vivo* redox state

The effects of PUFA on the redox state were initially investigated to determine the primary anti-aging effects of PUFA in the facet of the free radical theory of aging. Various antioxidase activities in selected tissues were investigated because of their critical antioxidative defense capacities. Moreover, malonaldehyde is considered as an oxidative stress biomarker, which is a typical end product of PUFA and expressed as TBARS equivalents to indicate the lipid peroxidation state in biological membranes [20].

**Figure 1:**
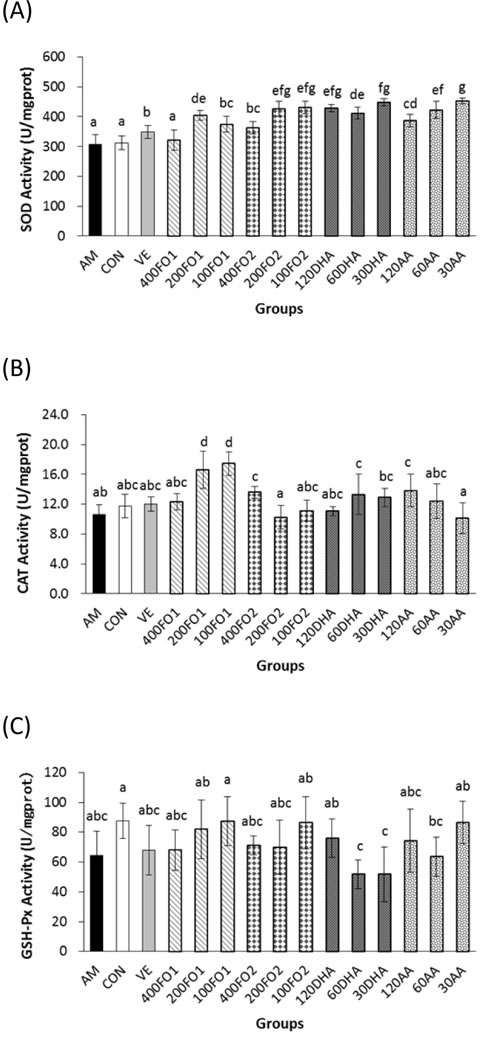
PUFA ameliorate hepatic oxidative stress Both fish oil and PUFA monomers significantly promoted hepatic **A**. SOD and **B**. CAT activities (*P* < 0.05). However, the improvement in **C**. GSH-Px activities did not seem significant (*P* > 0.05). Each column with error bar indicates the data expressed as mean ± standard deviation (SD) (*n* = 5). Bars marked with different letters indicate significant discrepancies (*P* < 0.05). AM, aging model group; Con, saline control group; VE, vitamin E positive control; 400FO1, 200FO1, and 100FO1, FO1 (DHA/EPA ratio = 2.28) at high, moderate and low doses, respectively; 400FO2, 200FO2, and 100FO2, FO2 (DHA/EPA ratio = 0.66) at high, moderate and low doses, respectively; 120DHA, 60DHA, and 30DHA, DHA at high, moderate and low doses, respectively; 120AA, 60AA, and 30AA, AA at high, moderate and low doses, respectively.

Compared with the aging model group, fish oil and DHA treatments in all groups except the high-dose No. 1 fish oil (400FO1) group significantly promoted hepatic SOD activities within the range of 18%-46% in a non-linear dose-response manner, whereas the arachidonic acid (AA) treatment enhanced activities in a dose-dependent manner (*P* < 0.05) (Figure 1A). FO1 at a moderate dose, FO2 at low and moderate doses and PUFA monomers at all doses were significantly superior to vitamin E for their promotion effects on hepatic SOD activities. Moreover, FO1 (100 and 200 mg/kg/d), FO2 (400 mg/kg/d), DHA (60 mg/kg/d) and AA (120 mg/kg/d) treatments improved hepatic catalase (CAT) activities by 65%, 56%, 28%, 26% and 31%, respectively (*P* < 0.05, Figure 1B). However, improvements of hepatic glutathione peroxidase (GSH-Px) activities were not identified in all groups (*P* > 0.05, Figure 1C). Similarly, previous work identified different improvements in these hepatic antioxidase activities [21], indicating antioxidase species may vary in the sensitivity to nutritional intervention of PUFA. Notably, fish oil had no significant effect on lipid peroxidation in the liver (*P* > 0.05, Supplementary Table S2), whereas PUFA monomers at high and moderate doses adversely increased the hepatic TBARS level (*P* < 0.05, Supplementary Table S2). This result indicates that PUFA monomers may exhibit a high oxidative potential due to their multiple conjugated double bonds.

Expectedly, the mice in aging model group exhibited significantly minimized cardiac SOD activities compared with the control group (*P* < 0.05). Fish oil and PUFA monomers promoted cardiac SOD activity within the range of 19%-58% in the aging mice (*P* < 0.05, Supplementary Figure S1). Interestingly, only FO1 at a moderate dose significantly reduced cardiac lipid peroxidation, whereas AA monomer at high and low doses increased the TBARS levels (*P* < 0.05, Supplementary Table S2), which indicates a side effect of AA monomer supplementation.

Furthermore, monoamine oxidase (MAO) also contributes to the ROS production because it catalyzes the oxidative deamination of dietary amines, monoamine neurotransmitters, and hormones that generate hydrogen peroxide [22]. Moreover, the age-related increase in MAO activity is acknowledged to contribute to brain cellular degeneration [23]. Therefore, we assessed the activities of MAO and SOD in the brain. As shown in Supplementary Figure S2, the reduced SOD activities and increased MAO activities in the brains of mice in the aging model group were consistent with the mimetic aging process *via* injection with D-galactose (*P* < 0.05). FO1 at all doses and FO2 at high and moderate doses ameliorated the reduction in cerebral SOD activities within the range of 27%-34% and 31%-38%, respectively, whereas AA at a moderate dose only enhanced SOD activities by 5% compared with the aging model group (*P* < 0.05, Supplementary Figure S2A). In addition, both fish oil and PUFA monomers were effective in inhibiting cerebral MAO activities (Supplementary Figure S2B) and TBARS levels (Supplementary Table S2) within the range of 56%-90% and 16%-62%, respectively (*P* < 0.05). Notably, DHA and AA monomers exhibited opposite dose-response effects in the inhibition of cerebral MAO activities. Briefly, these data indicate that PUFA may be beneficial for cerebral oxidative stress and have a protective potential for age-related degeneration.

Considering the challenging facet of ROS as extremely reactive and short-lived molecules, F_2_-isoprostane, which represents the gold standard for oxidative stress status *in vivo*, was measured. This prostaglandin F_2_-like compound is formed *in vivo* *via* non-enzymatic free radical-catalyzed peroxidation of AA [24]. In addition, the serum GSH-Px activities are investigated to determine the antioxidative defense. The results demonstrated that supplemental PUFA substantially reduced the *in vivo* oxidative stress status, as evidenced by significant inhibition of the plasma F_2_-isoprostane levels within the range of 25%-79% (*P* < 0.05, Supplementary Figure S3A). However, only high-dose FO2 significantly enhanced the serum GSH-Px activities (*P* < 0.05, Supplementary Figure S3B).

Taken together, both fish oil and PUFA monomers exerted promising regulation of the *in vivo* redox state *via* the promotion of antioxidase (hepatic CAT and SOD in the liver, heart and brain) activities and the reduction of cerebral MAO activities, cerebral TBARS levels and the plasma F_2_-isoprostane levels (*P* < 0.05). However, supplemental PUFA monomers at high and moderate doses may have the side effect of an increased TBARS level (*P* < 0.05). In addition, the dose-response manner of redox state improvement depends on the observed index. The different DHA/EPA ratios in FO1 and FO2 produced a minimized discrepancy in the *in vivo* redox state improvement, particularly the dose-response effects on the hepatic CAT, and cerebral SOD and MAO activities.

### n-3 rather than n-6 PUFA ameliorate telomere shortening

**Figure 2:**
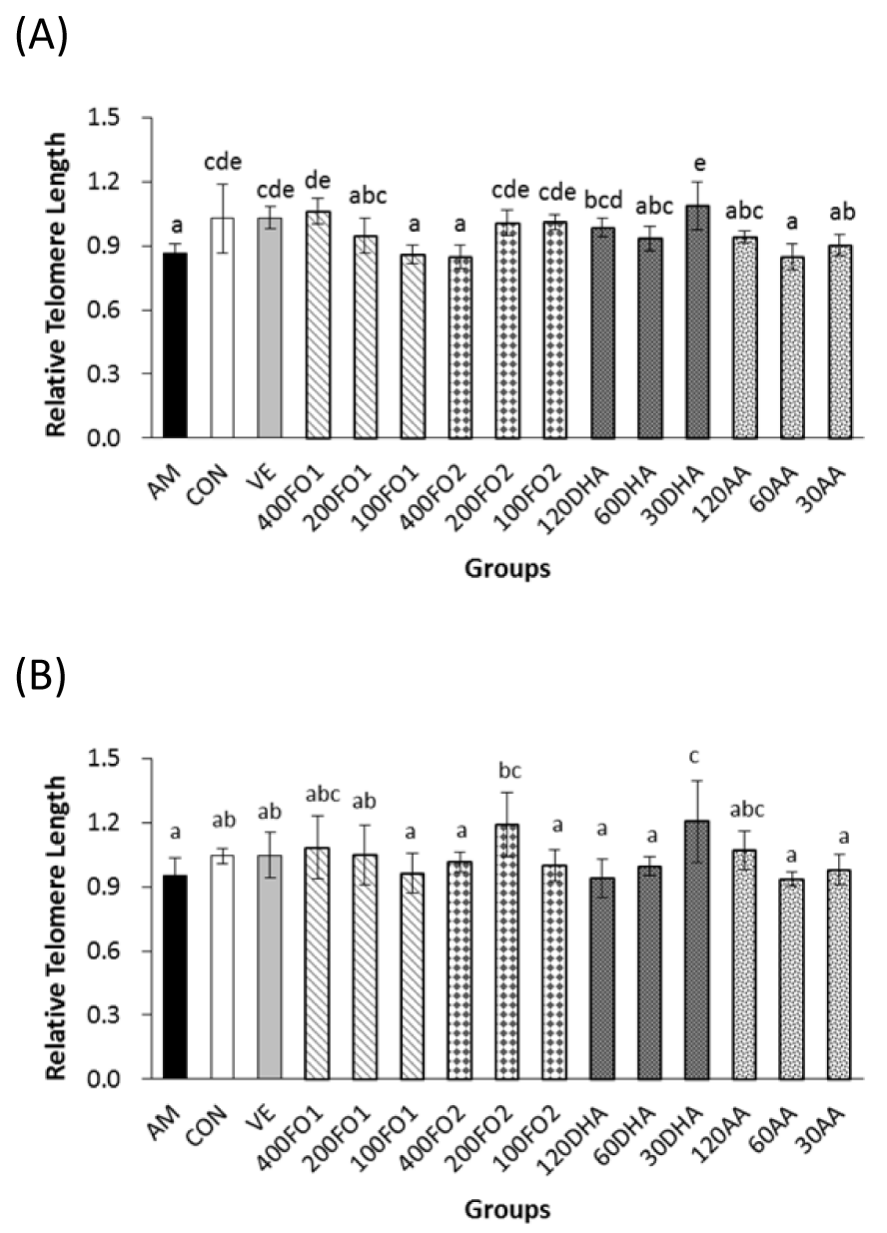
n-3 rather than n-6 PUFA inhibit excessive telomere length loss in **A**. livers and **B**. testes of aging mice. Each column with error bar indicates the data expressed as mean ± SD (*n* = 5). Bars marked with different letters indicate significant discrepancies (*P* < 0.05). The instructions about the group labels were the same as indicated in Figure 1.

The effects of PUFA on telomere attrition were subsequently investigated to indicate an intrinsic aging process. Expectedly, the hepatic telomere length of mice in the aging model group was significantly shorter than the control group (*P* < 0.05, Figure 2A), which was in accordance with the expectation that telomere erosion is accelerated in the D-galactose-induced aging model. Compared with the aging model group, the samples in high-dose FO1, low- and moderate-dose FO2, and high- and low-dose DHA groups significantly inhibited hepatic telomere shortening within the range of 13%-25% (*P* < 0.05), whereas no promising effect was identified in the AA groups (*P* > 0.05, Figure 2A). Telomere attrition in the testes was not as obvious as in the liver; nevertheless, moderate-dose FO2 and low-dose DHA exhibited 25% and 27% inhibitory effects on the testicular telomere attrition, respectively (*P* < 0.05, Figure 2B).

Briefly, n-3 rather than n-6 PUFA supplements effectively ameliorate telomere shortening in the format of an n-3 PUFA-enriched fish oil or monomer as exemplified in the telomere length analysis of the liver and testes (*P* < 0.05). Moreover, the telomere protected by n-3 PUFA does not appear in a linear dose-response manner.

### Both n-3 and n-6 PUFA inactivate testicular telomerase through the inhibition of *c-Myc*-mediated *TERT* expression

**Figure 3:**
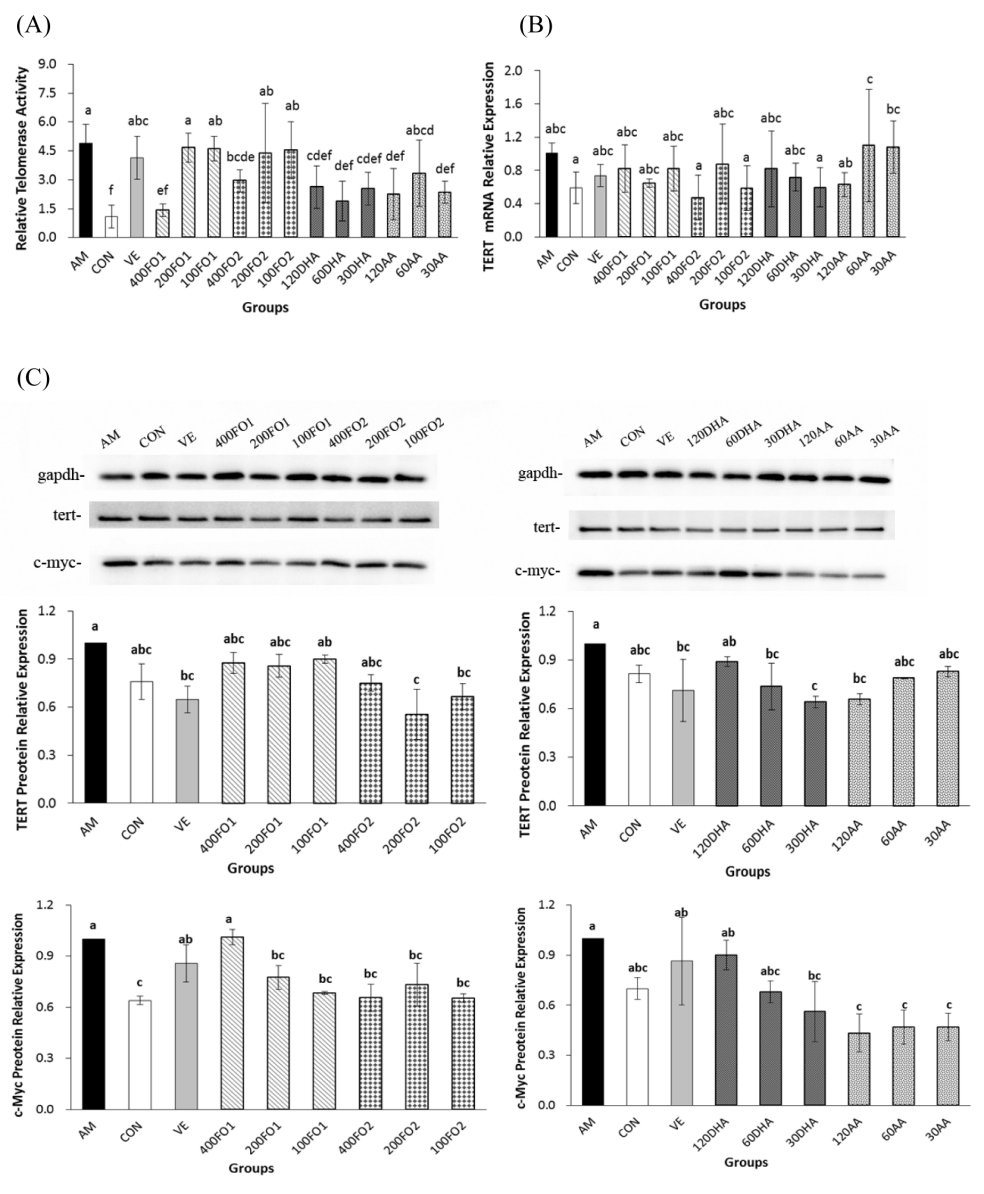
PUFA repress testicular telomerase activities through the reduction of ***c-Myc***-mediated ***TERT*** expression in aging mice. **A**. The effect of PUFA on telomerase activities (*n* = 5). **B**. The effect of PUFA on the mRNA expression of testicular *TERT* (*n* = 5). **C**. The effect of PUFA on the protein expression of testicular *TERT* and *c-Myc* (*n* = 3). The *TERT* and *c-Myc* primary antibody recognized a single protein band with molecular weights of approximately 126 kDa and 57 kDa, respectively. Each column with error bar indicates the data expressed as mean ± SD. Bars marked with different letters indicate significant discrepancies (*P* < 0.05). *gapdh*, glyceraldehyde-3-phosphate dehydrogenase. The instructions about the group labels were the same as indicated in Figure 1.

To further explain the telomere protection in the n-3 PUFA-supplemental groups, the testicular telomerase activities were measured. Representative images of the testes in the experimental groups were shown in Supplementary Figure S4. Surprisingly, the mice that exhibited shorter telomere in the aging model group demonstrated substantially higher telomerase activities compared with the control group (*P* < 0.05, Figure 3A). It has been reported that telomere length homeostasis required limiting telomerase levels [25]. Therefore, the explanation for ectopic telomerase expression in aging mice is likely attributed to the response induced by acute telomere erosion. Moreover, high-dose fish oil, all-dose DHA and high- and low-dose AA significantly repressed telomerase activities within the ranges of 40%-71%, 45%-61% and 52%-54%, respectively (*P* < 0.05, Figure 3A). Collectively, FO2 at a moderate dose exerted a telomere-protective effect, whereas a high dose was required to suppress telomerase activity. The low-dose DHA monomer was capable of protecting telomere erosion, whereas all-dose DHA monomer was effective on inactivating telomerase activity. Moreover, AA at all doses had no influence on the telomere length, but AA at high and low doses significantly inhibited telomerase activity. There was no significant difference of telomerase inhibition between n-3 and n-6 PUFA (*P* > 0.05).

**Figure 4:**
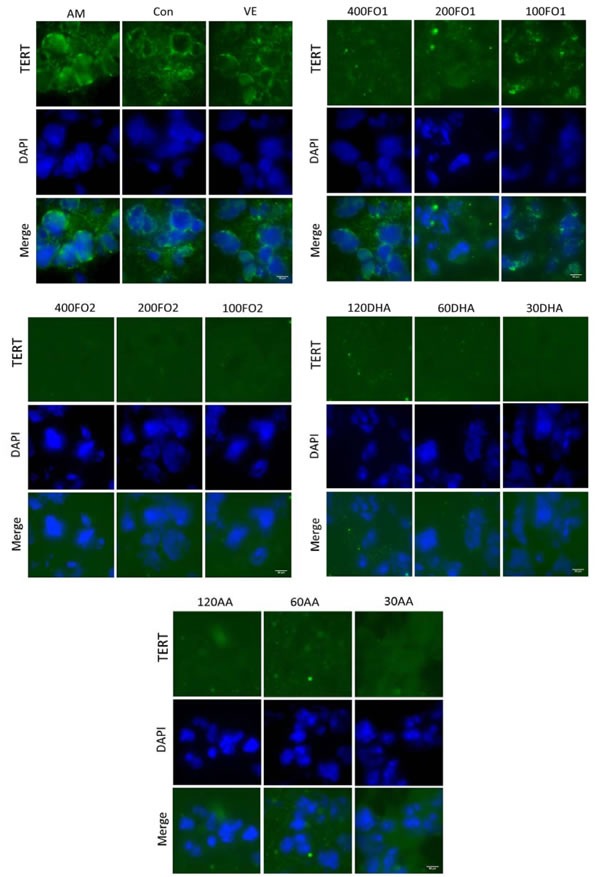
PUFA reduce the testicular ***TERT*** protein expression confirmed by the immunofluorescence of histological testicular sections. DAPI, 4’,6-diamidino-2- phenylindole. Scale bar, 50 μm.

To further confirm telomerase inhibition by PUFA, we subsequently analyzed the expression of *TERT* and its transcription factor, *c-Myc*, in the testes. *TERT*, which may be regulated both transcriptionally and epigenetically, is a catalytic subunit of telomerase and its expression is acknowledged as the rate-limiting factor for telomerase catalytic activity [26]. For example, *c-Myc* directly mediates *TERT* transcriptional activation because the *TERT* promoter contains numerous *c-Myc*-binding sites [27]. Figure 3B indicates that no significant change in the *TERT* mRNA expression was identified in the aging mice (*P* > 0.05). However, both FO2 and DHA at moderate and low doses and AA at high dose significantly reduced the *TERT* protein expression within the ranges of 25%-45%, 26%-36% and 34%, respectively (*P* < 0.05, Figure 3C). The discrepancy between the mRNA and protein expression of *TERT* remains enigmatic. Nevertheless, a similar inconsistency has been reported with regard to other genes [28]. One potential explanation is that complicated biological processes, including transcriptional or post-transcriptional splicing, translational modifications, and other processes, may alter the relative quantities of mRNA and protein to various degrees [28]. Furthermore, the protein expression of *c-Myc* was also significantly reduced within the range of 30%-60% following the intervention of moderate- and low-dose FO1, all-dose FO2, low-dose DHA, and all-dose AA (*P* < 0.05, Figure 3D). We further used immunofluorescence assay to demonstrate the above reduction effect of PUFA on the protein expression of *TERT* and *c-Myc* (Figure 4). Overall, telomerase inactivation by PUFA in aging mice may be explained by the *c-Myc*-mediated *TERT* pathway.

### DHA inhibits testicular antioncogene *p16* and *p53* expression

**Figure 5:**
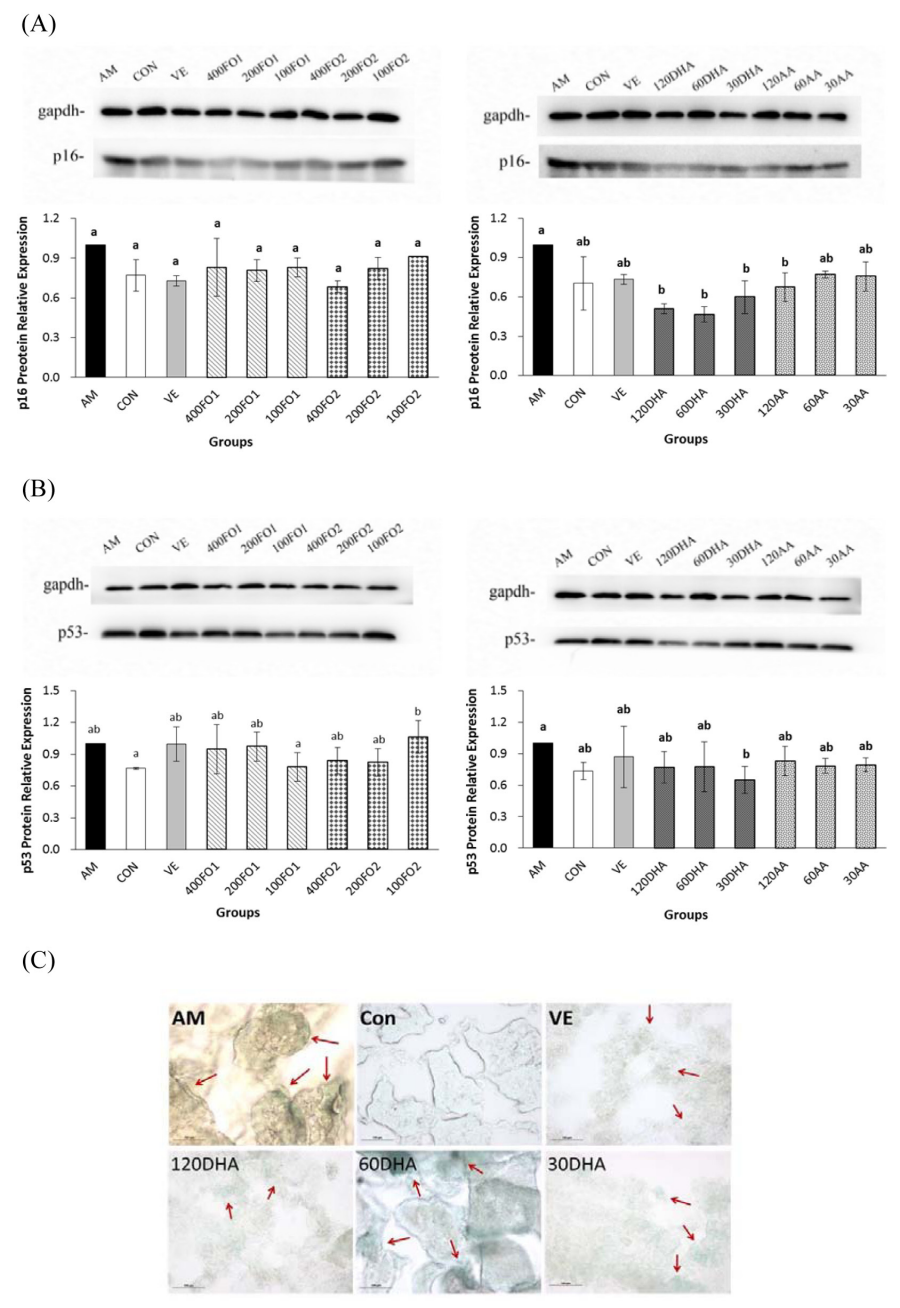
DHA inhibits testicular ***p16*** and ***p53*** protein expression and SA-β-gal. **A**. The effect of PUFA on the protein expression of testicular *p16*. **B**. The effect of PUFA on the protein expression of testicular *p53*. The *p16* and *p53* primary antibodies recognized a single protein band with molecular weights of approximately 16 kDa and 53 kDa, respectively. **C**. Representative fields of *in situ* SA-β-gal staining in testicular cryosections. Arrows indicate foci with high SA-β-gal activities. Each column with error bar indicates the data expressed as mean ± SD (*n* = 3). Bars marked with different letters indicate significant discrepancies (*P* < 0.05). The instructions about the group labels were the same as indicated in Figure 1.

Furthermore, effects of PUFA on the critical molecular pathways of cellular senescence in the testes of aging mice were investigated. The results indicated that all-dose DHA significantly inhibited the testicular *p16* protein expression and low-dose DHA repressed the *p53* protein expression (*P* < 0.05, Figure 5A and 5B), whereas fish oil did not significantly alter the *p16* and *p53* expression. The senescence-associated β-galactosidase (SA-β-gal) staining of whole-mount testes and testicular cryosections only indicated a mild discrepancy in the PUFA intervention groups (Supplementary Figure S5). However, DHA remained effective in the inhibition of SA-β-gal activities in the testicular cryosections of aging mice (Figure 5C).

Taken together, DHA rather than AA significantly inactivated the *p16* and *p53* expressions at their certain intervention levels. Combined with previous findings, we conclude that n-3 rather than n-6 PUFA may ameliorate the senescence process *via* the redox-telomere-antioncogene axis.

## DISCUSSION

Consistent with previous findings that fish oil and PUFA are capable of ameliorating oxidative stress in various tissues [29, 30], we demonstrated that both fish oil and PUFA monomers also effectively ameliorate D-galactose-induced oxidative stress *in vivo* by promoting antioxidase activities (mainly hepatic CAT and hepatic, cardiac and cerebral SOD activities) and reducing the plasma F_2_-isoprostane levels. In addition, the beneficial protection of PUFA against cerebral oxidative stress evidenced by reduced cerebral TBARS levels and MAO activities is consistent with previous work [31]. However, long-chain PUFA are susceptible to oxidation, and a high dietary intake of n-3 PUFA may trigger additional oxidative stress without adequate antioxidative protection. For example, the plasma TBARS levels were significantly increased in the fish oil group compared with the placebo control during pregnancy [32]. Supplemental fish oil increased the TBARS levels in autoimmune-prone NZB/W female mice fed *ad libitum* and injected with cyclophosphamide compared with corn oil [15]. In our present work, supplemental PUFA monomers at high and moderate doses promoted hepatic TBARS levels, whereas AA at high and low doses also increased cardiac oxidative stress. PUFA monomers without the presence of antioxidants are more susceptible to oxidation in free fatty acid forms than fish oil *in vivo*, which ultimately produces an increased TBARS level. Previous studies have demonstrated that n-3 PUFA were superior to n-6 PUFA in the enhancement of antioxidase activities [21, 33]. Interestingly, our findings indicated that DHA may promote antioxidase activities to the same extent as AA because DHA has more double bonds than AA and is more susceptible to oxidation. Therefore, the oxidative propensity of PUFA, as well as its functionality should be simultaneously taken into consideration.

With respect to the telomere theory of aging, several recent studies have provided epidemiological evidence that n-3 PUFA may prevent excessive telomere attrition [18, 19, 34]. Similarly, we demonstrated that only n-3 PUFA was effective in inhibiting hepatic and testicular telomere shortening in aging mice. In addition, telomere shortening depends on the balance between oxidative stress and antioxidative defense because telomeres are triple-G-containing structures, which are susceptible to damage by oxidative stress [35]. Therefore, our results revealed that the protective effect of n-3 PUFA on telomere may be partly attributed to the amelioration of redox state. Nevertheless, both n-3 and n-6 PUFA significantly reduced telomerase activities independent of the telomere length. This finding is probably associated with the two-edged-sword property of telomerase. Telomerase is canonically responsible for telomere length maintenance, whereas its activation may favor tumorigenesis [36]. Telomere dysfunction is believed to drive the early stages of cancer development, and subsequent telomerase activation appears to be critical for malignant progression [37]. Moreover, telomerase activity and telomere length have been acknowledged as markers for distinguishing prostate cancer from normal and benign prostate tissues [38]. In this context, both n-3 and n-6 PUFA are beneficial for testicular telomere hemostasis and protective from prostate cancer *via* the inhibition of ectopic telomerase expression in the aging model group. An *in vitro* study has demonstrated that EPA and DHA suppressed the telomerase activity and *TERT* mRNA levels in a time- and dose-dependent manner [39]. Thus, telomerase acts as a critical molecular target in the growth and survival of cancer cells modulated by n-3 PUFA [40]. Similarly, our results demonstrated that long-chain PUFA were effective in inactivating telomerase likely through the inhibition of *c-Myc*-mediated *TERT* expression.

Oxidative stress has been demonstrated to be a potent inducer of *p53* [2], which also acts in a context-dependent manner. To respond low levels of oxidative stress, *p53* exhibits antioxidant activities to ensure cell survival. In contrast, to respond severe oxidative stress, *p53* exhibits prooxidant activities and leads to cell death [41]. Therefore, it is not difficult to interpret the controversial data that n-3 PUFA may inhibit [42, 43] or induce [44, 45] *p53* expression in both *in vivo* and *in vitro* experiments. However, none of these studies have addressed the effects of n-3 PUFA on *p53* expression in an anti-aging view. Similarly, *p16* also has a balance in cancer and senescence [44, 46]. Many noxious stimuli, including ROS, ionizing radiation, and UV light, have been demonstrated to induce *p16* expression *in vitro* and *in vivo* [47]. The *p16* expression substantially increases in nearly all rodent tissues with advancing age. Thus, it is acknowledged as a robust biomarker of aging [11]. Precise expression of *p16* is essential for tissue homeostasis, maintaining a coordinated balance between tumor suppression and aging. Although anti-tumor drug development teams are in favor of activating the antioncogene *p16*, aging biologist groups aim to block the accumulation of *p16*-positive cells. As a result, the key to longevity likely depends on the hands of both groups, as a careful balance of *p16* expression is required to stave off cancer and prevent aging [48]. Such balance is also applicable when n-3 PUFA are used to manage antioncogenes. A recent study indicated the anti-aging potential of n-3 PUFA that the overexpression of *Fat1*, an endogenously synthesizing n-3 PUFA model, in Boer goat fetal fibroblasts reduced the mRNA expression of *p16* and *p53* [49]. Nevertheless, fish oil mediates apoptosis of tumor cells by promoting the protein expression of *p53* [50]. In accordance with this study, our results demonstrated the inhibition of both *p16* and *p53* protein expression by DHA, which contributes to the hypothesis that n-3 PUFA may slow down the process of cell and organ senescence.

Additionally, our present work demonstrated that both n-3 and n-6 PUFA inactivated telomerase, thereby exerting their anti-cancer potential. However, DHA significantly inhibited *p16* and *p53*, which appears vague in the setting of tumor inhibition. *In vivo* studies have demonstrated that the inhibition of tumor suppressors (such as *p16* and *p53*) and up-regulation of telomerase activity and *TERT* expression may promote tumorigenesis, including prostate cancer [51, 52], whereas the opposite features underline senescence [53]. Therefore, both n-3 and n-6 PUFA may inhibit tumorigenesis *via* the suppression of oxidative stress-induced telomerase activation, whereas n-3 PUFA exerted an anti-aging effect *via* the protection of telomere attrition and down-regulation of tumor suppressors (Supplementary Figure S6).

The reason for DHA rather than fish oil effectively inhibiting the protein expression of *p16* and *p53* may be ascribed to the multi-ingredient mixture of fish oil including DHA, EPA, monounsaturated fat and other compounds. In addition to n-3 PUFA compositions, ethyl esters and triglycerides are two main forms of commercial fish oil available in the market [54]. The apparent bioavailability is considered to be the lowest for the ethyl ester form and the highest for the free fatty acid form [55]. Thus, the anti-aging effect of PUFA in different conjugation forms requires further investigations.

In conclusion, we systematically investigated the *in vivo* anti-aging effect of PUFA from the facets of oxidative stress amelioration and telomere protection. Long-chain PUFA effectively ameliorated the *in vivo* redox state in aging mice *via* promoting SOD activity and reducing cerebral MAO activity, cerebral TBARS levels and plasma F_2_-isoprostane levels. However, supplemental PUFA monomers at high and moderate doses should be carefully administered in cases of increased oxidative stress. In addition, both n-3 and n-6 PUFA significantly inhibited telomerase activities due to the reduction of *c-Myc*-mediated *TERT* expression, which indicates the tumor-inhibitive potential of long-chain PUFA. However, only n-3 PUFA protected telomere from progressive attrition, and DHA was superior in the inhibition of tumor suppressors, *p16* and *p53*, which indicates that n-3 PUFA may be served as a potent anti-aging supplement.

## MATERIALS AND METHODS

### Animals and experimental design

One hundred twenty Institute of Cancer Research (ICR) male mice (8 weeks old, weighed 30-40 g), purchased from the Laboratory Animal Research Center of Zhejiang Chinese Medical University, were housed at (20±1) °C on a cycle of 12-h light/12-h dark. All experimental protocols were approved by the Ethical Committee of the College of Biosystems Engineering and Food Science at Zhejiang University in China. Mice were randomly assigned to 15 groups (8 animals per group): the negative saline control (Group Con), the D-galactose-induced aging model (Group AM), the vitamin E positive control (Group VE), and groups of FO1 (Groups 400FO1, 200FO1, and 100FO1), FO2 (Groups 400FO2, 200FO2, and 100FO2), DHA (Groups 120DHA, 60DHA, and 30DHA) and AA (Groups 120AA, 60AA, and 30AA) with high, moderate and low administration doses. The mice in the negative control group were intraperitoneally injected with vehicle (saline) throughout the two-month experiment, whereas the mice in all other groups were injected with D-galactose (Aladdin-Reagent Co. Ltd., Shanghai, China) solution at a dose of 80 mg/kg/d to induce senescence. The mice in the Con and AM groups were administered 100 mg/kg/d of corn oil, whereas the mice in the VE group received 100 mg/kg/d of vitamin E. To observe the possible dose-response anti-aging effect of PUFA, the mice in FO1 and FO2 groups received 400, 200 and 100 mg/kg/d of fish oil *via* oral gavage, while the mice in DHA and AA groups were orally administered with 120, 60 and 30 mg/kg/d of DHA and AA, respectively. Both the intraperitoneal injection and oral gavage were performed daily. The mice in each group were weighed weekly to adjust the dose of the D-galactose and treatment samples.

### Determination of antioxidase activities

The hepatic, cerebral and cardiac SOD activities, hepatic CAT activity, hepatic and serum GSH-Px activities, and cerebral MAO activity were determined using kits (Nanjing Jiancheng Bioengineering Institute, Nanjing, China) according to the manufacturer's instructions.

### Determination of TBARS levels

The TBARS in the liver, heart and brain were determined using the well-established thiobarbituric acid test with a kit (Nanjing Jiancheng Bioengineering Institute, Nanjing, China).

### Determination of F_2_-isoprostane levels

The plasma F_2_-isoprostane levels were measured using an enzyme immunoassay kit (Cayman Chemical, Ann Arbor, MI, USA).

### Telomere length analysis

The relative telomere length in the liver and testicle, expressed as a ratio of the telomere repeat copy number to single-copy gene copy number (*T/S*), was measured *via* quantitative real-time polymerase chain reaction (PCR) as previously described, while *36B4* (acidic ribosomal phosphoprotein PO) is acknowledged as the single-copy gene [56]. The sequences and final concentrations of the primers (Invitrogen Life Sciences, Carlsbad, CA) were as follows: Tel F, 5’-CGGTTTGTTTGGGTTTGGGTTTGGGTTTGGGTT TGGGTT-3’, 300 nM; Tel R, 5’-GGCTTGCCT TACCCTTACCCTTACCCTTACCCTTACCCT-3’, 900 nM; 36B4 F, 5’-ACT GGT CTA GGA CCC GAG AAG-3’, 300 nM; and 36B4 R, 5’-TCA ATG GTG CCT CTG GAG ATT-3’, 500 nM.

### Telomerase activity assay

The testicular telomerase activities in all groups were measured using a TeloTAGGG telomerase PCR ELISA kit (Roche applied science, Indianapolis, IN, USA).

### Real-time quantitative reverse transcription PCR

RNA was isolated using TRIzol reagent (Invitrogen Life Technologies, Carlsbad, CA) from frozen testicle samples and reverse transcribed into cDNA using a RT reagent kit with gDNA Eraser (Takara Bio Inc., Otsu, Japan) according to the manufacturer's instructions. The sequences and final concentrations of the primers for *mTERT* and β-actin (as the internal control) were as follows: β-actin F, 5’-TGACATCCGTAAAGA-3’, 400 nM; β-actin R, 5’-CAGCTCAGTAACAGTCC-3’, 400 nM; mTERT F, 5’-ATGGCGTTCCTGAGTATG-3’, 400 nM; mTERT R, 5’-TTCAACCGCAAGACCGACAG-3’, 400 nM. Similarly, the ΔΔ*C*t method (mTERT /β-actin = 2^−ΔΔCt^) was used to analyze the results.

### Whole-mount and *in situ* SA-β-gal staining

Whole-mount testes SA-β-gal staining and *in situ* SA-β-gal staining in testicular cryosections were processed with a Senescence β-Galactosidase Staining Kit (Cell Signaling Technology, Beverly, USA) with modifications.

### Western blotting analysis and immunofluorescence

Standard Western blotting and immunofluorescence procedures were conducted with modifications. The primary antibodies for detecting protein expression included rabbit polyclonal anti-β-actin (as the internal control; E021070; EarthOx, LLC, San Francisco, CA), rabbit polyclonal anti-TERT (sc-7212; Santa Cruz Bio., Santa Cruz, CA), rabbit monoclonal anti-c-Myc (#5605; Cell Signaling Technology, Beverly, MA), mouse monoclonal anti-p53 (ab26; Abcam, Cambridge, MA) and mouse monoclonal anti-p16 (SAB5300498, Sigma-Aldrich, St. Louis, MO). Secondary antibodies purchased from EarthOx, LLC (San Francisco, CA) and the anti-goat Alexa Fluor 488-conjugated secondary antibody (Invitrogen Life Technologies, Carlsbad, CA) were used in the Western blotting analysis and immunofluorescence, respectively.

### Statistical analysis

Statistical analysis was conducted with SPSS version 16.0 software, and all data were presented as the mean ± SD. Multiple comparisons among different groups were conducted with a one-way analysis of variance (ANOVA) followed by Duncan's test. Data were considered statistically significant at *P* < 0.05.

Detailed procedures of the above experiments were shown in Supplementary Materials and Methods.

## SUPPLEMENTARY MATERIALS FIGURES AND TABLES


