# Supplementary material for: Polyunsaturated fatty acids ameliorate aging via redox-telomere-antioncogene axis
Source: Oncotarget. 2016 Dec 26;8(5):7301–14. doi: 10.18632/oncotarget.14236 (PMC5352322; doi:10.18632/oncotarget.14236)
Supplement: Supplementary file 1 [file oncotarget-08-7301-s001.pdf]

Polyunsaturated fatty acids ameliorate aging *via* redox-telomere-antioncogene axis

Supplementary Information

This supplementary information includes six supplementary figures, two supplementary tables and supplementary materials and methods.

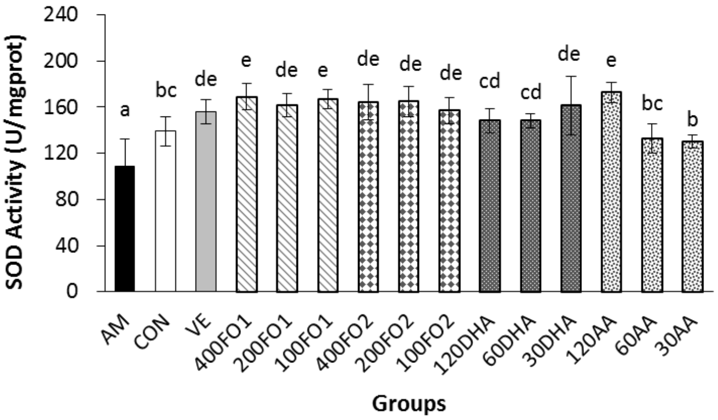

Supplementary Figure S1: PUFA ameliorate cardiac oxidative stress in aging mice. Both fish oil and PUFA monomers significantly promoted cardiac SOD activities (P<0.05). Each column with error bar indicates the data expressed as mean ± standard deviation (SD) (n=5). Bars marked with different letters indicate significant discrepancies (P<0.05). AM, aging model group; Con, saline control group; VE, vitamin E positive control; 400FO1, 200FO1, and 100FO1, FO1 (DHA/EPA ratio=2.28) at high, moderate and low doses, respectively; 400FO2, 200FO2, and 100FO2, FO2 (DHA/EPA ratio=0.66) at high, moderate and low doses, respectively; 120DHA, 60DHA, and 30DHA, DHA at high, moderate and low doses, respectively; 120AA, 60AA, and 30AA, AA at high, moderate and low doses, respectively.

(A)

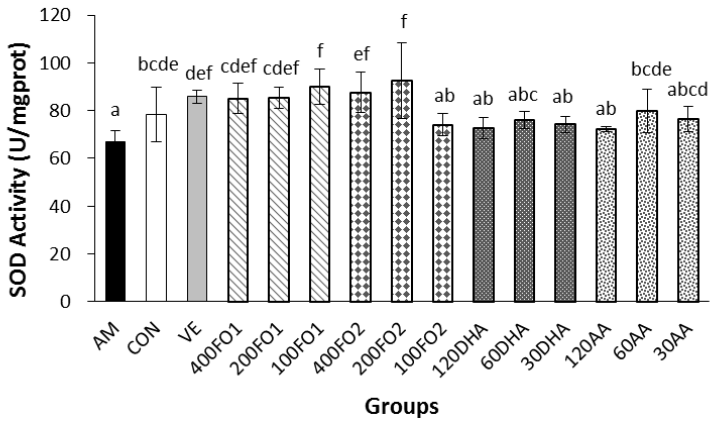

(B)

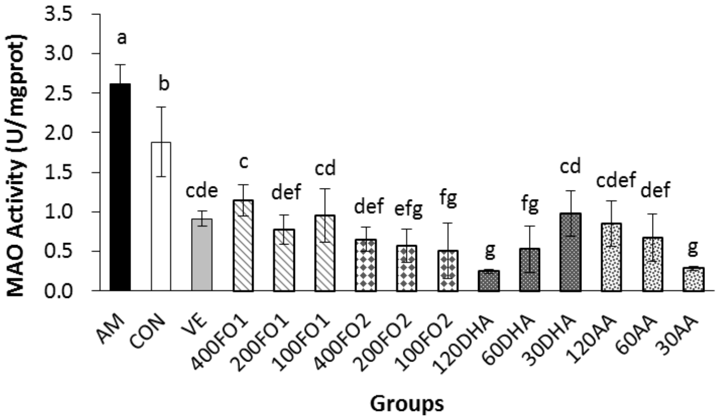

Supplementary Figure S2: PUFA ameliorate cerebral oxidative stress in aging mice. (A) Fish oil at all doses and AA at moderate dose significantly promoted cerebral SOD activities. (B) Both fish oil and PUFA monomers significantly inhibited MAO activities (*P*<0.05). Each column with error bar indicates the data expressed as mean ± SD (*n*=5). Bars marked with different letters indicate significant discrepancies (*P*<0.05). The instructions about the group labels were the same as indicated in Supplementary Figure S1.

(A)

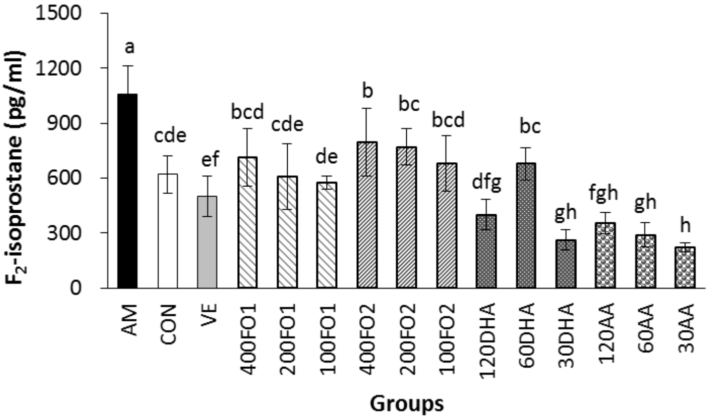

(B)

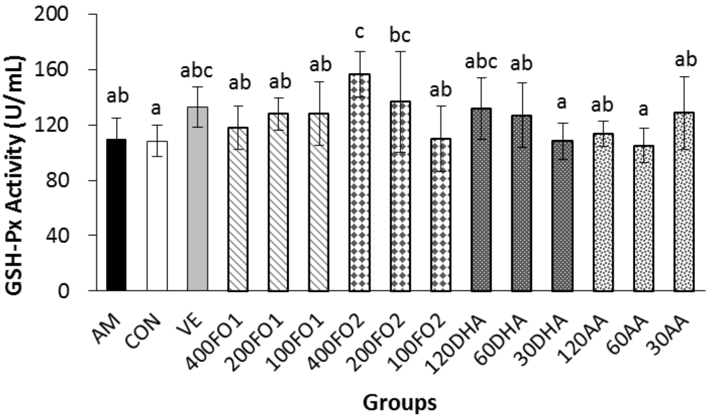

Supplementary Figure S3: PUFA ameliorate overall oxidative stress in aging mice. (A) Both fish oil and PUFA monomers significantly reduced plasma F_2-isoprostane levels, whereas (B) only FO2 at high dose promoted serum GSH-Px activities (P<0.05). Each column with error bar indicates the data expressed as mean ± SD (n=5). Bars marked with different letters indicate significant discrepancies (P<0.05). The instructions about the group labels were the same as indicated in Supplementary Figure S1.

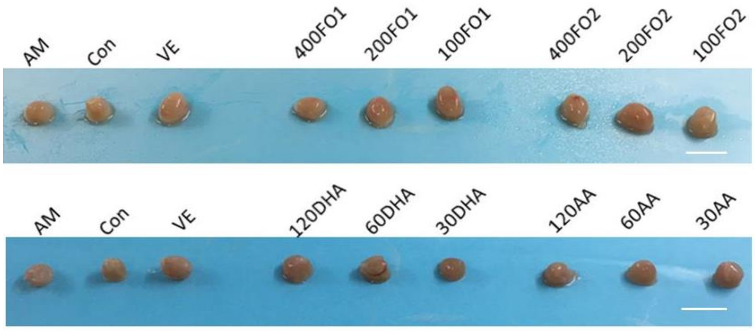

Supplementary Figure S4: Representative images of testes in all experimental groups. No significant atrophy was identified. Scale bar, 1 cm. The instructions about the group labels were the same as indicated in Supplementary Figure S1.

(A)

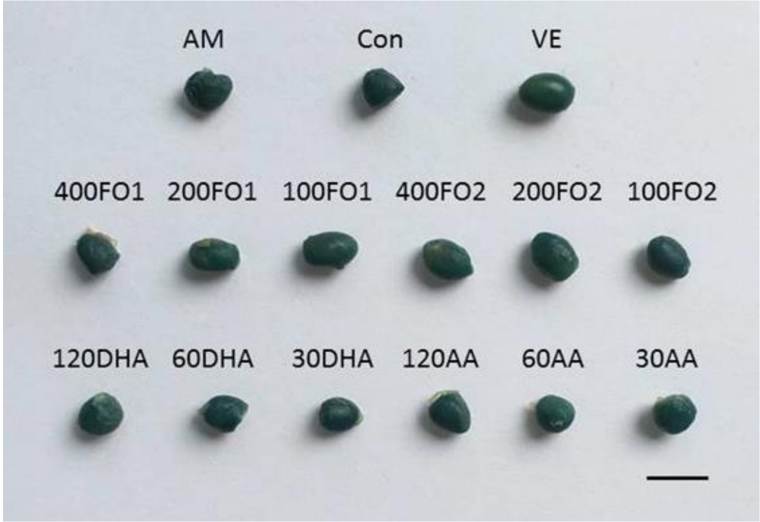

(B)

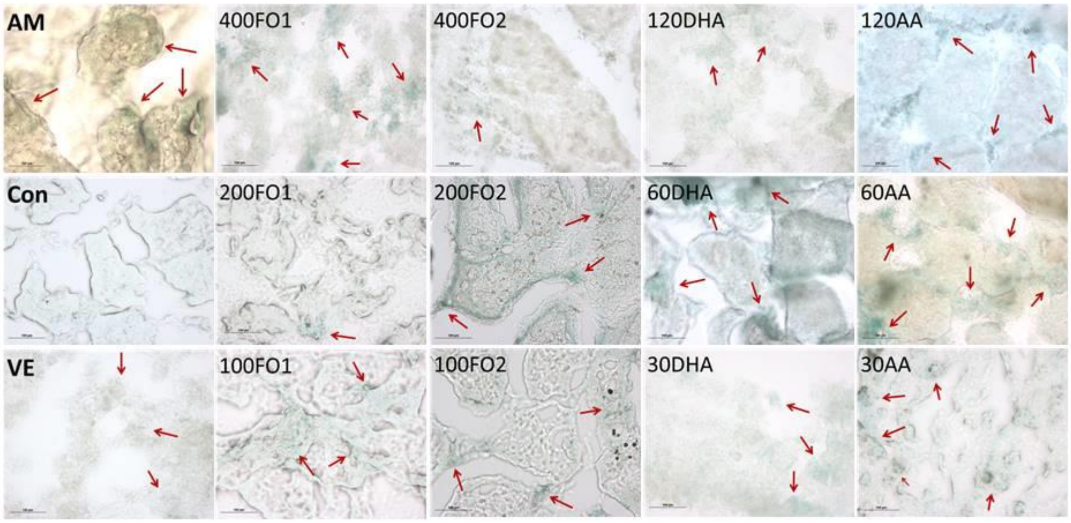

Supplementary Figure S5: (A) Whole-mount testes SA-β-gal staining and (B) in situ SA-β-gal staining in testicular cryosections indicated few differences in the PUFA intervention groups, whereas the senescence-associated beta-galactosidase activities were minimal in the testicular cryosections of the Con and VE groups. The instructions about the group labels were the same as indicated in Supplementary Figure S1.

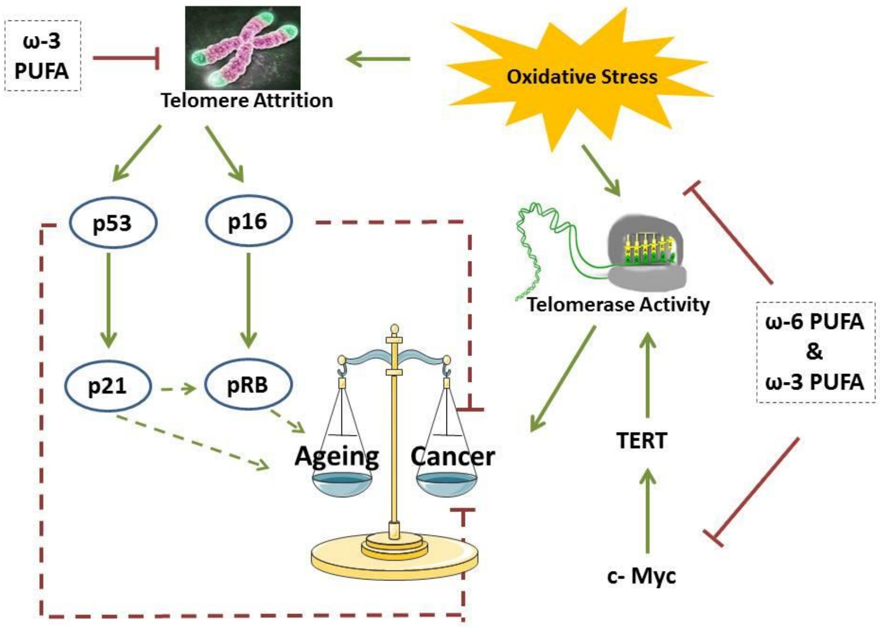

Supplementary Figure S6: The balance of aging and cancer may be regulated by PUFA in the testes of aging mice. Oxidative stress increased the tumorigenesis propensity via the activation of telomerase activities, which were inhibited by both n-6 and n-3 PUFA through a reduction in *c-Myc*-mediated *TERT* protein expression. Furthermore, oxidative stress induced excessive telomere attrition and tumor suppressor expression, which were ameliorated by n-3 PUFA.

Supplementary Table S1: Body weight of aging mice

Group [*]	Body weight (g) [†]	
	Baseline	8 week
AM	35.54±1.97	41.36±0.88
Con	38.09±2.07	44.39±3.10 *
VE	36.76±2.37	39.96±2.38
400FO1	38.25±1.44	43.03±2.62
200FO1	36.88±3.19	40.80±4.26
100FO1	36.86±1.55	44.21±2.77
400FO2	35.04±2.15	43.48±1.39
200FO2	37.89±2.58	44.56±2.83 *
100FO2	36.34±0.98	42.76±2.75
120DHA	36.24±3.51	40.33±4.34
60DHA	35.38±2.60	41.51±3.21
30DHA	37.24±1.64	43.21±2.81
120AA	36.34±2.17	41.55±2.51
60AA	36.82±2.58	41.36±2.07
30AA	37.30±3.64	41.94±4.37

[*] The instructions about the group labels were the same as indicated in Supplementary Figure S1.

[†] Data were expressed as mean ± SD (n=8). Data were considered statistically significant compared with the AM group. * P<0.05.

Supplementary Table S2: Effects of PUFA on lipid peroxidation in the liver, heart and brain of aging mice

Groups [*]	TBARS level ± SD (nmol/mgprot) [†]		
	Liver	Heart	Brain
AM	0.94 ±0.14 [abc]	2.29 ±0.22 [bcde]	2.83 ±0.67 [a]
Con	0.75 ±0.50 [ab]	2.01 ±0.13 [abc]	1.27 ±0.20 [bc]
VE	0.59 ±0.06 [a]	1.90 ±0.04 [abc]	1.38 ±0.20 [bcd]
400FO1	0.57 ±0.04 [a]	1.70 ±0.07 [ab]	2.07±0.38 [fg]
200FO1	0.76 ±0.59 [ab]	1.44 ±0.21 [a]	1.08 ±0.21 [b]
100FO1	1.13 ±0.15 [abcd]	1.65 ±0.29 [ab]	1.55 ±0.73 [cde]
400FO2	0.96 ±0.09 [abc]	1.88 ±0.29 [abc]	1.58 ±0.28 [cde]
200FO2	1.25 ±0.05 [bcd]	2.30 ±0.31 [bcde]	1.78 ±0.16 [def]
100FO2	1.29 ±0.24 [bcd]	7.87 ±1.61 [h]	1.78 ±0.18 [def]
120DHA	3.18 ±0.96 [fg]	2.06 ±0.54 [abcd]	1.50 ±0.30 [bcde]
60DHA	2.52 ±0.85 [e]	2.82 ±0.55 [def]	2.96 ±0.66 [a]
30DHA	1.46 ±0.24 [cd]	2.65 ±0.36 [cde]	2.37 ±0.20 [g]
120AA	1.58 ±0.20 [d]	5.12 ±0.70 [g]	1.88 ±0.41 [ef]
60AA	3.41 ±0.85 [g]	2.99 ±0.85 [ef]	1.35 ±0.31 [bcd]
30AA	1.05 ±0.15 [a]	3.46 ±0.84 [f]	1.30 ±0.28 [bc]

[*] The instructions about the group labels were the same as indicated in Supplementary Figure S1.

[†] Data were expressed as mean ± SD (n=5). Different letters indicate significant discrepancies (P<0.05).

Supplementary Materials and Methods

Preparation of sample solutions

The contents of Deep Sea Fish Oil Soft Capsules (HongtaoK Group Holding Co., Ltd., Wuhan, Hubei, China), Liquid Softgel Fish Oil (Nature Made, Mission Hills, CA, USA) and Natural Vitamin E Capsules (Conba Pharmaceutical Co., Ltd., Hangzhou, Zhejiang, China) were collected to obtain fish oil No.1 (FO1), No.2 (FO2) and vitamin E, respectively. FO1 and FO2 were analyzed via gas chromatography (GC) using the 7890A gas chromatograph equipped with a flame ionization detector (Agilent, Santa Clara, CA, USA) according to a previously published work [1]. A close constituent of monounsaturated fat (20%), total carbohydrate (20%), protein (less than 20%) and antioxidants (negligible) was present in FO1 and FO2, which was inferred from the supplement facts. Therefore, the major difference between FO1 and FO2 is the ratio of docosahexenoic acid (DHA) to eicosapentenoic acid (EPA), which were determined by GC and calculated as 2.28 and 0.66 for FO1 and FO2, respectively. DHA and arachidonic acid (AA) standard samples (Sigma-Aldrich, St. Louis, MO) were subsequently dissolved in a small amount of edible corn oil (Yihaikerry Group Co., Ltd., Shanghai, China) for the preparation of solutions with gradient concentrations and were stored in nitrogen-filled glass tubes at 4 °C to prevent oxidation. Fresh aliquots were prepared once per week in the following animal study.

Animals and experimental design

One hundred twenty Institute of Cancer Research (ICR) male mice (8 weeks old, weighed 30-40 g, Kunming strain), purchased from the Laboratory Animal Research Center of Zhejiang Chinese Medical University, were housed at (20±1) °C on a cycle of 12-h light/12-h dark. All experimental protocols were approved by the Ethical Committee of the College of Biosystems Engineering and Food Science at Zhejiang University, China. Mice were randomly assigned to 15 groups (8 animals per group):

the negative saline control (Group Con), the D-galactose-induced aging model (Group AM), the vitamin E positive control (Group VE), and groups of FO1 (Groups 400FO1, 200FO1, and 100FO1), FO2 (Groups 400FO2, 200FO2, and 100FO2), DHA (Groups 120DHA, 60DHA, and 30DHA) and AA (Groups 120AA, 60AA, and 30AA) with high, moderate and low administration doses. The mice in the negative control group were intraperitoneally injected with vehicle (saline) throughout the two-month experiment, whereas the mice in all other groups were injected with a D-galactose (Aladdin-Reagent Co. Ltd., Shanghai, China) solution at a dose of 80 mg/kg/d to induce senescence. The mice in the Con and AM groups were administered 100 mg/kg/d of corn oil, whereas the mice in the VE group received 100 mg/kg/d of vitamin E. To observe the dose-response fashion in the anti-aging effect of PUFA, the mice in the FO1 and FO2 groups received 400, 200 and 100 mg/kg/d of fish oil via oral gavage, and the mice in the DHA and AA groups were orally administered 120, 60 and 30 mg/kg/d of DHA or AA monomer. Both the intraperitoneal injection and oral gavage were performed daily. The mice in each group were weighed weekly to adjust the dose of the D-galactose and treatment samples.

Preparation of blood and tissues

The femoral arterial blood of 8-h fasted mice was collected, and the mice were subsequently sacrificed to obtain their livers, hearts and brains. These tissues were shock-frozen in liquid nitrogen after collection and stored at -80 °C until analysis.

The serum and plasma isolated from the collected blood samples were used for the determination of antioxidase activities and F_2-isoprostane levels, respectively. The tissue preparation for the determination of antioxidase activity was performed as follows. A piece (approx. 100 mg) was cut from the frozen tissue sample and transferred to a sterile micro-centrifuge tube. Following the addition of pre-cooled saline (1 mL per 100 mg of tissue), the tissue sample was subsequently homogenized (IKA-T10 homogenizer; IKA, Staufen, Germany) on ice until a uniform consistency was achieved. The mixture was then centrifuged (500 ×g) at 4 °C for 10 min to collect

the supernatant.

Determination of antioxidase activities

Superoxide dismutase (SOD) activities in the brain, liver and heart were assessed using a SOD detection kit (Nanjing Jiancheng Bioengineering Institute, Nanjing, China). Briefly, the superoxide radicals generated from the xanthine-xanthine oxidase (XOD) system reduce the formation of a water-soluble tetrazolium salt (WST-1) formazan, which may be inhibited by SOD from the supernatants of the samples and spectrophotometrically measured at 550 nm. The SOD activities were calculated as follows, in which 50% represents the inhibition rate. One unit of SOD activity is defined as the amount of protein that inhibits half of the WST-1 formazan formation. The tissue SOD activities were expressed as U/mg protein.

$$\text{Tissue SOD activity} = \frac{(\text{blank OD} - \text{sample OD}) \times \text{dilution factor}}{\text{blank OD} \times (\text{sample protein conc.} \times 50\%)}$$

The hepatic catalase (CAT) activities were determined according to the manufacturer’s instructions (Nanjing Jiancheng Bioengineering Institute, Nanjing, China). Briefly, the supernatants from homogenized livers were treated with sufficient H_2O_2 for degradation catalyzed by CAT, and the remaining H_2O_2 composited with ammonium molybdate to produce a maximum absorption at 405 nm. The CAT activities were calculated as follows, in which 271 represents the slope reciprocal of the standard curve and 60 represents a dilution factor. The hepatic CAT activities were expressed as U/mg protein.

$$\text{Hepatic CAT activity} = \frac{(\text{blank OD} - \text{sample OD}) \times 271}{\text{sample amount} \times \text{sample protein conc.} \times 60}$$

The hepatic and serum glutathione peroxidase (GSH-Px) activities were determined using a kit (Nanjing Jiancheng Bioengineering Institute, Nanjing, China).

The principle is that GSH-Px catalyzes the oxidation of GSH in the presence of H_2O_2, and the remaining GSH may be quantified using a reaction with 5,5'-dithio-bis-(2-nitrobenzoic acid) (DTNB) at 422 nm. The GSH-Px activities were calculated as follows, in which 20 represents the standard concentration (μmol/L). The hepatic and serum GSH-Px activities were expressed as U/mg protein and U/mL, respectively.

$$\text{Hepatic GSH} - \text{Px activity} = \frac{(\text{nonenzymatic OD} - \text{enzymatic OD}) \times 20 \times \text{dilution factor}}{(\text{standard OD} - \text{blank OD}) \times (\text{reaction time} \times \text{sample amount} \times \text{sample protein conc.})}$$

$$\text{Serum GSH} - \text{Px activity} = \frac{(\text{nonenzymatic OD} - \text{enzymatic OD}) \times 20 \times \text{dilution factor}}{\text{standard OD} - \text{blank OD}}$$

Determination of monoamine oxidase (MAO) activities

Brain MAO activities were evaluated with a kit (Nanjing Jiancheng Bioengineering Institute, Nanjing, China). Briefly, benzaldehyde formation using benzylamine as the substrate is catalyzed by MAO, and benzaldehyde extracted by cyclohexane exhibits a maximum absorbance at 242 nm. The MAO activities were calculated as follows, and one unit of MAO activity is defined as per milligram of protein generating 0.01 OD value. The brain MAO activities were expressed as U/mg protein.

$$\text{Brain MAO activity} = \frac{\text{sample OD}}{0.01 \times \text{reaction time} \times \text{sample amount} \times \text{sample protein conc.}}$$

Determination of thiobarbituric acid reactive substances (TBARS) levels

The TBARS contents in the liver, heart and brain were determined using a well-established thiobarbituric acid (TBA) test with a kit (Nanjing Jiancheng

Bioengineering Institute, Nanjing, China). Malonaldehyde (MDA) and related compounds in the supernatants of the samples may react with TBA in acidic media at 100 °C, and the reaction mixture is spectrophotometrically measured at 532 nm. Therefore, the TBARS level is typically adapted to assess the MDA level. The MDA levels were calculated as follows, in which 10 represents the standard concentration (μmol/L). The results were expressed as nmol/mg protein.

$$\text{Tissue TBARS level} = \frac{\text{sample OD} - \text{blank OD}}{(\text{standard OD} - \text{blank OD}) \times \text{sample protein conc.} \times 10}$$

Determination of F_2-isoprostane levels

The plasma F_2-isoprostane levels were measured with an enzyme immunoassay (EIA) kit (Cayman Chemical, Ann Arbor, MI, USA). Briefly, a diluted sample (1:5, v/v) was incubated with the F_2-isoprostane tracer and rabbit antiserum overnight at 4 °C. The amount of F_2-isoprostane tracer bound to antiserum is inversely proportional to the F_2-isoprostane level in the sample. Followed by quintuple washes, the samples were incubated with Ellman's reagent. Finally, the development of yellow color was measured at 412 nm using a microplate reader (Varioskan Flash, Thermo Fisher Scientific Inc., Waltham, MA, USA) [2]. The plasma F_2-isoprostane levels were expressed as pg/mL following standard calibration.

Telomere length (TL) analysis

The relative TL in the liver and testes, which was expressed as a ratio of the telomere repeat (T) copy number to the single-copy gene (S) copy number (*T/S*), was measured via quantitative real-time polymerase chain reaction (PCR) as previously described. The *36B4* (acidic ribosomal phosphoprotein PO) gene is well accepted as the single-copy gene [3]. Briefly, genomic DNA was extracted from the liver and testes according to the instructions of the Mammalian Genomic DNA Miniprep Kit (Sigma, St Louis, MO, USA). The purity and concentration of the isolated DNA were determined using a NanoDrop 2000 spectrophotometer (Thermo Scientific,

Wilmington, DE, USA).

For PCR reactions, two separate plates were used to specifically amplify T or S. Each sample assayed for both T and S amplifications was analyzed in quadruple. Therefore, four identical 2-μL aliquots of the DNA sample (20 ng) were added into one plate, and an additional four aliquots were added into the same well position in another plate. For each standard curve, a reference DNA sample was serially diluted in TE buffer (10 mM Tris-HCl, 0.1 mM EDTA, pH 7.5) by two fold per dilution to produce eight concentrations of DNA that ranged 0.395-50 ng/μL. Three identical 2 μL of each concentration were distributed to the standard curve wells on each plate. The reference DNA sample to which all experimental samples were compared was a pooled sample from isolated DNA samples from all groups. In addition, the 20 mL-PCR reaction system also contained 10 μL SYBR Premix Ex Taq II (Takara Bio Inc., Otsu, Japan), 0.4 μL ROX Reference Dye II (Takara Bio Inc., Otsu, Japan), T or S primers and sterile double-distilled water. The sequences and final concentrations of the primers (Invitrogen Life Sciences, Carlsbad, CA) were as follows: Tel F, 5'-CGGTTTGTTTGGGTTTGGGTTTGGGTTTGGGTTTGGGTT-3', 300 nM; Tel R, 5'-GGCT TGCCTTACCCTTACCCTTACCCTTACCCTTACCCT-3', 900 nM; 36B4 F, 5'- ACT GGT CTA GGA CCC GAG AAG -3', 300 nM; and 36B4 R, 5'- TCA ATG GTG CCT CTG GAG ATT -3', 500 nM. The PCR reaction was conducted on a 7500 Fast Real-Time PCR System (Applied Biosystems, Foster City, CA) in a 96-well format, with the following thermal cycling profile: 95 °C incubation for 30 seconds, followed by 40 cycles of 95 °C for 5 seconds, 55 °C for 30 seconds, and 72 °C for 30 seconds. The samples were normalized to single-copy genes as indicated, and the fold change was calculated using the ΔΔ*C*t method ($T/S = 2^{-\Delta\Delta Ct}$).

Telomerase activity assay

The testicular telomerase activities in all groups were measured using a TeloTAGGG telomerase PCR ELISA kit (Roche applied science, Indianapolis, IN, USA) according to the manufacturer's instructions. Following determination of the

protein concentration with a Bradford Protein Assay (Bio-rad Laboratories, Hercules, CA), 9 μg protein were added to the reaction mixture to participate in the telomeric repeat amplification protocol (TRAP) assay [4]. The amplified PCR product was subsequently subjected to treatment for the enzyme-linked immunosorbent assay, and the color change was detected using a microplate reader (Varioskan Flash, Thermo Fisher Scientific Inc., Waltham, MA, USA). The relative activity was measured compared with the lysis buffer control.

Real-time quantitative reverse transcription PCR

RNA was isolated using TRIzol reagent (Invitrogen Life Technologies, Carlsbad, CA) from frozen testicle samples and reverse transcribed into cDNA using a RT reagent kit with gDNA Eraser (Takara Bio Inc., Otsu, Japan) according to the manufacturer's instructions. With the same thermal cycling profile as previously described, quantitative real-time PCR on cDNA was performed in triplicate using SYBR Premix Ex Taq II (Takara Bio Inc., Otsu, Japan) on a CFX96 Real-Time PCR Detection System (Bio-Rad Laboratories, Hercules, CA). The sequences and final concentrations of the primers for mTERT and β-actin (as the internal control) were as follows: β-actin F, 5'-TGACATCCGTAAAGA-3', 400 nM; β-actin R, 5'-CAGCTCAGTAACAGTCC-3', 400 nM; mTERT F, 5'-ATGGCGTTCCTGAGTATG -3', 400 nM; mTERT R, 5'-TTCAACCGCAAGACCGACAG-3', 400 nM. Similarly, the ΔΔCt method (mTERT /β-actin = $2^{-\Delta\Delta Ct}$) was used to analyze the results.

Whole-mount and in situ SA-β-gal staining

Whole-mount testes SA-β-gal staining and in situ SA-β-gal staining in testicular cryosections were processed with a Senescence β-Galactosidase Staining Kit (Cell Signaling Technology, Beverly, USA) with modifications. Thawed mouse testes were fixed in 1 × Fixation Buffer supplied from the kit for 90 min. After washing with PBS three times, the testes were incubated at 37 °C for 16 h with a staining mixture that contains pH 6.0 buffer and X-gal substrate. The images were captured using a NIKON

SMZ18 microscope (Nikon, Tokyo, Japan).

Western blotting analysis

Sections from the frozen testicle samples were prepared in RIPA Lysis Buffer (Millipore, Temecula, CA) with Protease inhibitors (Fdbio Science, Hangzhou, China) and subsequently homogenized (IKA-T10 homogenizer; IKA, Staufen, Germany) on ice until there was a uniform consistency. Following a 30-minute incubation on ice, the lysate was centrifuged at 13,500 × g for 20 minutes at 4 °C. The protein concentration in the removed supernatant was determined as previously described. A standard western blotting procedure was subsequently conducted as previously described [5] with modifications. Briefly, 25-35 μg aliquots of total protein were electrophoresed, transferred onto a PVDF membrane (Millipore, Temecula, CA) and probed with specific primary antibodies (1:1000). With the binding of HRP-conjugated antibodies, the bands were visualized using ECL (Fdbio Science, Hangzhou, China) on a G:BOX Chemi XX6 imaging system (Syngene, Cambridge, UK). Grayscale densitometry for blot quantification was performed using ImageJ software (U.S. National Institutes of Health, Bethesda, MD, USA), which was normalized by the internal control β-actin. The primary antibodies for the detection of protein expression included rabbit polyclonal anti-β-actin (E021070; EarthOx, LLC, San Francisco, CA, USA), rabbit polyclonal anti-TERT (sc-7212; Santa Cruz Bio., Santa Cruz, CA, USA), rabbit monoclonal anti-c-Myc (#5605; Cell Signaling Technology, Beverly, MA), mouse monoclonal anti-p53 (ab26; Abcam, Cambridge, MA) and mouse monoclonal anti-p16 (SAB5300498, Sigma-Aldrich, St. Louis, MO). The second antibodies were purchased from EarthOx, LLC (San Francisco, CA, USA).

Immunofluorescence

Frozen testes sections (~8 μm) were fixed with formaldehyde for 10 min at room temperature and permeabilized with 0.1% triton X-100 (v/v) in PBS for 4 min at

room temperature, followed by a block with 1% BSA (w/v) in PBS for 1 hour at room temperature. The sections were subsequently incubated with rabbit polyclonal anti-TERT antibody (sc-7212; Santa Cruz Bio., Santa Cruz, CA, USA; 1:50) overnight at 4 °C, followed by incubation with anti-goat Alexa Fluor 488-conjugated secondary antibody (Invitrogen Life Technologies, Carlsbad, CA; 1:1000) for 1 h at room temperature. After the last wash with PBS-Triton (0.1%), the nuclei were stained with DAPI (Beyotime, Haimen, China; 1:10000) for 5 min prior to mounting with glycerol jelly mounting medium (Beyotime, Haimen, China). Images were captured on an N-SIM super-resolution microscope system (Nikon, Tokyo, Japan) and cropped in NIS-Elements C software.

Supplementary References

1. Kang JX, Wang J. A simplified method for analysis of polyunsaturated fatty acids. BMC Biochem. 2005; 6:5.
2. Labuschagne CF, van den Broek NJF, Postma P, Berger R, Brenkman AB. A protocol for quantifying lipid peroxidation in cellular systems by F2-isoprostane analysis. PLoS One. 2013; 8:e80935.
3. Cawthon RM. Telomere measurement by quantitative PCR. Nucleic Acids Res. 2002; 30:e47.
4. Kim NW, Wu F. Advances in quantification and characterization of telomerase activity by the telomeric repeat amplification protocol (TRAP). Nucleic Acids Res. 1997; 25:2595-2597.
5. Hirano S. (2012). Western Blot Analysis. In: Reineke J, ed. Nanotoxicity: Methods and Protocols. (Totowa, NJ: Humana Press), pp. 87-97.
